# ADIDAS: An Examined Approach for Enhancing Cognitive Load and Attitudes towards Synchronous Digital Learning Amid and Post COVID-19 Pandemic

**DOI:** 10.3390/ijerph192416972

**Published:** 2022-12-17

**Authors:** Mostafa Aboulnour Salem, Abu Elnasr E. Sobaih

**Affiliations:** 1 Deanship of Development and Quality Assurance, King Faisal University, Al-Ahsa 31982, Saudi Arabia; 2 Management Department, College of Business Administration, King Faisal University, Al-Ahsa 31982, Saudi Arabia; 3 Hotel Management Department, Faculty of Tourism and Hotel Management, Helwan University, Cairo 12612, Egypt

**Keywords:** cognitive load theory, cognitive load, synchronous digital learning, distance learning, instructional design, SARS-CoV-2, COVID-19, ADIDAS model

## Abstract

SARS-CoV-2 (COVID-19) has disrupted university education and turned it into distance learning for at least one semester in many countries, including the Kingdom of Saudi Arabia (KSA). However, there was an issue with university students’ cognitive load at this critical time, because education totally stopped for about a month and then resumed remotely. This research draws on the cognitive load theory, particularly the extraneous load, to develop an instructional design model called ADIDAS. The model includes six stages, namely: analyse (A), design (D), improve (I), do (D), Assess (A), and Share (S). Thirty-four experts in instructional technology models have reviewed the ADIDAS model in Arab university contexts, producing a consensus about its suitability for use in distance learning amid the COVID-19 pandemic. Following the consensus of the experts, the model was applied to a sample of 527 students at King Faisal University, KSA. The results confirmed significant statistical differences with a very large effect size in relation to the attitude towards synchronous digital learning (SDL) and cognitive load pre and post ADIDAS. Students had a positive attitude towards SDL and a low cognitive load during the educational process pre adoption of the ADIDAS model, compared to post ADIDAS. The current research results have numerous implications for post the COVID-19 pandemic, especially in Arab countries and similar contexts.

## 1. Introduction

Due to the impact of SARS-CoV-2 (COVID-19), education in the Kingdom of Saudi Arabia (KSA), like many other nations worldwide, has shifted from traditional learning, i.e., face-to-face, to synchronous digital learning (SDL), with virtual classrooms using numerous tools, e.g., Blackboard Collaborate, Microsoft Teams, Zoom, and social media in some cases [1,2,3]. Before COVID-19, in the KSA, utilizing SDL in the higher education context was limited to some courses [4,5]. Furthermore, numerous studies have highlighted the various benefits of SDL: enhancing learning outcomes, learning satisfaction, learning performance, learning motivation, and positive attitudes towards education [6,7,8]. Amid COVID-19, in the first quarter of 2020, in a short time, the Ministry of Education in the KSA instructed all universities to turn all traditional classroom courses from a face-to-face setting to an online one [9]. Due to the sudden shift to SDL, educators were forced to utilise SDL as the sole tool for teaching and learning communication with their students [10]. Thus, this became a daunting job for educators who had presented and developed their courses remotely with individual efforts [11].

Accordingly, digital learning was expected to significantly improve cognitive achievement, skills, competencies, attitudes, and learning outcomes for higher education students during COVID-19 [12]. Conversely, recent studies in many countries showed adverse SDL influences amid COVID-19 among educators and students in higher education; for example, in the Philippines, students experienced different drawbacks to learning online issues such as low cognitive achievement, insufficient skills, and negative attitudes [13,14]. In China, research results showed a significant lack of students’ extrinsic motivation, intrinsic motivation, and deep cognitive engagement [15,16]. In Pakistan, educators experienced various constraints in executing their duties and responsibilities amid COVID-19, which affected learning outcomes [17,18]. In India, the usage of SDL during the COVID-19 pandemic has increased the lack of skills, time management, the lack of infrastructure/resources, poor communication at various levels, negative attitude, and student engagement [19]. In Saudi Arabia, despite the availability of an excellent technological infrastructure, investigators have reported on several of the adverse influences of SDL among higher education students amid COVID-19: low self-efficacy, lack of engagement and motivation, negative attitudes, cognitive load, and absence of goals [4,20,21]; also, they students mainly concerned about passing exams amid this educational situation [22].

Moreover, several studies [23,24] showed that synchronous digital learning (SDL) is often undertaken without fully considering the students’ cognitive load, which may lead to decreased competencies, the inability to retain and understand information, the absence of mental goals, and increased negative attitudes. Correspondingly, cognitive load depends on the chosen method of presenting information, student motivation, student involvement, and academic concern [25]. According to Chandler and Sweller [26], cognitive load refers to the method in which cognitive mental resources are focused and employed through learning and problem solving. Skulmowski and Xu [27] added that cognitive load is a consequence of the learning path and the critical factors determining the learning outcomes. Therefore, a student feels cognitive load when they cannot process the information and knowledge received during learning [28]. Hence, understanding occurs when all learning elements related to the educational contents are processed simultaneously by the working memory [26]. Consequently, if the educational contents have multiple factors that cannot be processed simultaneously in the working memory, the learning situation becomes challenging and is not understood, and cognitive load is formed [29]. Thus, higher education educators should enhance mental load distribution during SDL [30].

In addition, there are two categories of cognitive load: intrinsic and extraneous; both have implications for teaching, and educators can avoid the former and reduce the latter [26,27]. Moreover, intrinsic cognitive load (ICL) refers to the number of learning elements and the degree of interactivity required by the learning materials [29]; just how many cognitive features must be processed simultaneously for schema construction or element interactivity depends on the relational complexity of the learning content and a learner’s schema [28]. In contrast, extraneous cognitive load (ECL) is caused by an unneeded increase in the number of learning elements that must be processed simultaneously in the working memory [27]. Moreover, most of the articles on cognitive load theory have focused on the fact that cognitive load can be reduced by organizing vast amounts of previous information from the long-term memory to reduce the burden on the working memory and, thus, lower cognitive load [27,31]. Moreover, recent articles have pointed out that the cognitive load theory deals with aspects of learning and problem-solving difficulty that instructional design models can control [32,33]. In addition, instructional design models (IDMs) aim to optimise learning processes [34]. Thus, the central role of IDMs is processing learners’ knowledge construction individually, depending on the simple tasks to be solved [32]. However, IDMs are based on the given goals, learning tasks, learning materials, and activation of the learner’s cognitive processes to invest effort into learning [35,36]. Therefore, the current article focused on the proposed instructional design model (ADIDAS) to enhance learning, eliminate redundant materials, and arrange information to avoid splitting the learner’s attention, through which it is possible to reduce the cognitive load of higher education students during SDL.

Instructional design is the science and art of developing, evaluating, and maintaining learning situations that facilitate and improve teaching performance using IDMs [37]. In addition, numerous studies have benefited from instructional design during the COVID-19 pandemic [32,35]. Hence, adopting instructional design models is still one of the best practices to be mastered by higher education educators, especially during online learning amid global health emergencies, i.e., COVID-19 [38,39]. The approach of educational design has gradually evolved, observing a shift from a traditional prescriptive, normative definition to the current one based on diverse systems that are antipathetic to unique recipes and stringent specifications [35]. IDMs direct organized planning for learning elements (contents, activities, assessments, and others) [39]. Additionally, IDMs need a symmetry of sense and intuition, momentum to perform, and an ability to reflect on the accepted actions [29]. In general, all instructional design approaches depend on (at least) five significant steps: (1) analysis of the setting and student needs; (2) design of a bunch of specifications for an influential and applicable learning environment; (3) development of contents, activities, assess, and learners; (4) implementation of learning methods and strategies; (5) evaluation of all results [34,36,40]. In typical situations, besides the educators, the implementation of instructional design requires the presence of at least two people: (1) the instructional designer who designs the storyboard and (2) the e-learning developer who converts the developed storyboard into a product [34].

Many articles have examined the multiple IDMs that educators can use to deliver SDL amid COVID-19 [32,35,37]. According to Sangsawang [32], university educators’ adoption of IDMs could prioritise their students’ needs, feelings, and challenges with the IDMs’ designs during the transition amid COVID-19. According to Wang [37], utilising an instructional design model enhances adaptability and good planning, emphasising what it takes for an educator to serve their students. According to Hanafi, Yusuf et al. [41] and Xie [42], university educators’ adoption of IDMs improved students’ satisfaction with distance learning and enriched courses through extra activities. Though several studies have been conducted on IDMs and cognitive load among higher education students, especially in the context of Arab counties such as KSA, a lack of studies have been undertaken regarding educators’ utilisation of instructional design to address cognitive load among higher education students during synchronous digital learning amid COVID-19. 

The current study has two main objectives. First, it develops a new instructional design model for higher education educators to create positive attitudes towards SDL and enhance cognitive load amid global health emergencies, i.e., COVID-19. This novel instructional model is called ADIDAS, which will be discussed later, and has been examined by experts. Second, the study also examines the newly developed model with a sample of undergraduate students and compares their attitudes towards SDL and cognitive load pre and post ADIDAS. Therefore, the current study endeavours to answer two main questions. First, what is the ADIDAS model structure that educators can adopt for SDL amid emergencies, i.e., COVID-19? Second, to what extent does the ADIDAS model make a difference in attitudes towards SDL and cognitive load among higher education students amid emergencies, i.e., COVID-19, compared to pre-pandemic circumstances? 

Our hypotheses are that (1) there are significant differences in cognitive load among higher education students, pre and post ADIDAS-model adoption amid COVID-19, and (2) there are significant differences in attitudes towards SDL among higher education students, pre and post ADIDAS-model adoption amid COVID-19.

## 2. Methodology

### 2.1. Development of ADIDAS Model

To develop a draft of the ADIDAS model and answer the first research question, the literature review related to digital learning, especially amid COVID-19, instructional design models, and cognitive load as well as learning theories from various databases that we have reviewed and analysed (see Appendix A). After screening the literature, we focused our analysis on the ADDIE model [43], interaction analysis model (IAM) [44], and model of self-regulated learning (SRL) [45] to develop the first draft of the ADIDAS model.

The ADDIE model is a systematic, practical heuristic framework for synchronous online learning course development, which opens perfect possibilities and gives good outcomes. The IAM is one of the most frequently used instruments in the study of knowledge construction, and the extent of its use makes it one of the most coherent. Models of SRL depict learning as an activity that a learner self-regulates, whether learning alone (unsupervised) or in the presence of instructors or peers (supervised). These models were chosen to develop the first draft of ADIDAS. These models helped us to develop the ADIDAS model to achieve SDL goals, making it more interactive. They helped us apply the educational theories through SDL and optimal investment of learning elements, making the learner focus and rely on their efforts, raising the learner’s motivation, and providing sufficient space for the learner to interact with the learning elements. This motivates the learner to be creative and innovate and, thus, able to achieve comprehensive evaluation. Additionally, the ADIDAS model was based on the cognitive load theory [26]. Cognitive load theory ensures that learners acquire sufficient information and have secure dealing with novel information not to cause cognitive overload. It also considered Piaget’s cognitive constructivist theory [45] and the sociocultural constructivist learning theory [46].

The ADIDAS model was directed to 50 expert specialists in instructional technology, digital learning, cognitive psychology, and information technology in Middle Eastern countries. The purpose of this was to examine the validity of utilizing SDL. It is worth noting that responses were collected from only 34 experts, as 16 did not respond. The profile of these respondents is shown in Table 1. Experts were identified through personal networks and recommendations from different colleagues. 

**Figure 1:**
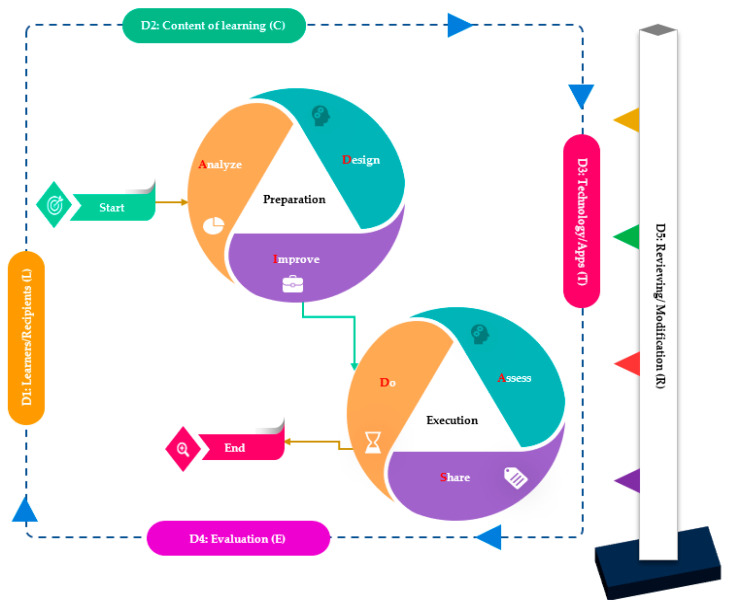
The ADIDAS model.

The procedures yielded 49 items with five dimensions, guiding the learning practices that help educators to provide SDL with low cognitive load. Each dimension of six constructs contained four factors: D1: learners/recipients (L), D2: the content of learning (C), D3: technology/apps (T), D4: evaluation (E), and D5: reviewing/modification (R). The 49 items that were included within the ADIDAS model for representing six constructs: Analyse (A) (9 items, α = 0.854); Design (D) (8 items, α = 0.862); Improve (I) (8 items, α = 0.799); Do (D) (10 items, α = 0.857); Assess (A) (9 items, α = 0.7986); Share (S) (5 items, α = 0.814). (See Figure 1 and Table 2). In Table 2, we explain the dimensions of the models and factors of each dimension as well as the items (with their original sources).

The ADIDAS model was circulated via experts’ private e-mails and social media on 1 October 2020 and maintained for three weeks. Day to day, the investigators reviewed and observed the responses. Cronbach’s alpha coefficient was used to assess the ADIDAS factors’ reliability.

### 2.2. Cognitive Load Scale

The procedures yielded 16 items with three factors measuring the students’ cognitive load. The scale showed good reliability and was derived from [29,30,48]. The 16 items that comprised the questionnaire for the study represent three factors: main cognitive load (6 items, α = 0.871), extraneous cognitive load (5 items, α = 0.793), and closely related cognitive load (5 items, α = 0.801). Each item was operationalised on a five-interval Likert scale, with students selecting one of five options to indicate the degree to which they reflect their cognitive load.

### 2.3. Attitude Scale

The procedures yielded 10 items with three factors measuring the students’ attitudes towards synchronous digital learning (SDL). The scale showed good reliability and was derived from [49,50]. The 10 items that comprised the questionnaire for the study represent three factors: knowledge development (3 items, α = 0.779), skills development (3 items, α = 0.832), and learning attitudes (4 items, α = 0.824). Each item was operationalised on a five-interval Likert scale, with students selecting one of five options to indicate the degree to which they reflect their attitude.

### 2.4. Research Population and Sample

The research population included all university students enlisted in King Faisal University (KFU) colleges in Al-Ahsaa, Eastern Province, KSA. The colleges at KFU relied considerably on online platforms and virtual classrooms to manage content, lectures, and exams amid the COVID-19 pandemic. Thirteen educators at King Faisal University were trained to utilise the ADIDAS model for SDL. The research team targeted 600 participating students, concerning their perceptions of cognitive load and attitudes towards SDL before and after using the ADIDAS model. According to Hill [51], the sample size calculation must be based on the total number of items, which should be at least five responses for each item. The items used in the current study were 49; hence, the sample should not be less than 245 responses. Furthermore, Muthén [52] added that a sample should be more than 150. In this research, our sample size was appropriate, since there were 527 valid responses for analysis. 

In total, 527 valid responses from students were received. Most students in the group were female (77.61% females, 22.39% males). Most respondents were between 18 and 20 (98.4%), and 6.7% of students had never used technology as an educational tool.

The educators provided the surveys to students via their private networks, i.e., WhatsApp, email, etc.. There was no power bias or authority over students. They were informed that the survey was just for scientific research and that their responses would be unidentified. Participants were voluntary and unnamed, and all the essential safeguards were utilised on site to assure data confidentiality. All personally identifiable information about participants was removed from the publicly available analysis, to ensure that answers could not be recognised. Further, distinct items such as name, age, etc., were optional.

### 2.5. Data Collection and Analysis

This research adopted a quantitative research methodology to develop the model and examine its effects on students’ cognitive load and attitudes towards SDL. The experts’ responses regarding the model were analysed and are presented in Table 1. Descriptive statistics were used to analyse the profile as well as cognitive load and attitudes towards SDL items. The responses of students, in relation to students’ cognitive load and attitudes towards SDL before and after the adoption of the ADIDAS model, were analysed by paired sample *t*-test using SPSS version 25. Eta squared was adopted to test the effect size. This gives a sign of the size of the variances between pre- and post-ADIDAS model adoption. 

## 3. Results

### 3.1. Students’ Cognitive Load 

To answer the second research question and examine the first research hypothesis, a comparison was made between pre- and post-ADIDAS adoption. First, descriptive statistics was adopted to analyse this using mean and standard deviation (Table 3). The results showed that the mean for pre-model adoption was between 1.403 (S.D. 0.586) and 1.504 (S.D. 0.616). On the other side, the mean for post-model adoption was between 4.184 (S.D. 0.917) and 4.534 (S.D. 0.784). These results show a significant difference between the pre- and post-ADIDAS model, which will be examined using the paired sample *t*-test.

Table 4 compares the three domains of cognitive load (CL): main (CL), extraneous (CL), and closely related (CL) pre and post implementation of the ADIDAS model. The results of the paired sample *t*-test showed significant differences in the three cognitive loads: main (CL), extraneous (CL), and closely related (CL) between pre-model and post-model adoption. Students achieved better results and had lower levels in the three domains of cognitive load after the implementation of the ADIDAS model. Students’ cognitive load (CL) pre-model adoption has the information presented density, traditional teaching methods, provided unnecessary information, and non-related activities; these did not contribute to the learning process. The effect size was very large, as confirmed by eta squared (Table 4); this means that the difference between pre- and post-model adoption was very large. Indeed, the better results were for post-model adoption (see Table 4). This supports the first research hypothesis (H1). 

### 3.2. Students’ Attitude towards Synchronous Digital Learning

To answer the second research question and examine the second research hypothesis, a comparison was undertaken between pre- and post-model adoption. First, descriptive statistics was adopted to analyse this using mean and standard deviation (Table 5). The results showed that the mean for pre-model adoption varies between 1.452 (S.D. 0.570) and 1.474 (S.D. 0.609). Conversely, the mean post-model adoption varies between 4.272 (S.D. 0.883) and 4.283 (S.D. 0. 893). These results show a significant difference between pre- and post-model adoption, which will be examined using the paired sample *t*-test.

Table 6 compares attitudes towards synchronous digital learning (SDL): knowledge development, skills development, and learning attitudes before and after the implementation of the ADIDAS model. The paired sample *t*-test showed significant differences in students’ attitudes towards SDL between pre-model and post-model adoptions. After implementing the ADIDAS model, students’ attitude synchronous digital learning SDL was positive compared to pre-model adoption. There was a lot of improvement in knowledge development, skills development, and learning attitude post-model adoption, compared to pre-model adoption. The effect size was very large, as confirmed by eta squared (Table 6); this means that the difference between pre- and post-model adoption was very large. Indeed, better results in attitude towards SDL were posted for the model adoption. This supports the second research hypothesis (H2).

## 4. Discussions

The current study was set to achieve two main objectives. Firstly, it was set to develop an instructional design model to create positive attitudes towards SDL and enhance cognitive load among higher education students amid COVID-19. Secondly, it examined the newly developed model with a sample of undergraduate students and compared their attitudes towards SDL and cognitive load pre and post adoption of the new model. The study was conducted on undergraduate students at King Faisal University, Saudi Arabia, amid the COVID-19 pandemic. Overall, the results showed significant statistical differences in cognitive load and attitude towards SDL post adoption of the ADIDAS model compared to pre-model adoption. These results are consistent with previous studies [25,53], which show that cognitive load and attitude towards SDL depend on the chosen method of presenting information, student motivation, and involvement in the learning process. 

The results confirmed that SDL amid the COVID-19 pandemic, as provided by the ADIDAS model, enriched the educational environment. The ADIDAS model provided appropriate methods for presenting and organising content, which reduced students’ cognitive load and increased levels of mental achievement, consistent with previous studies [25,47]. The ADIDAS model was based on the ADDIE model, which has been built according to the cognitive load theory; the information display methods and diversity of SDL gave the learners the necessary information to exclude redundant and repetitive activities unrelated to the content, which are regular in studies [28,31]. Furthermore, the transformation of learners from mere recipients of information contributed to alleviating the accidental cognitive load on working memory, increasing the learning process, and continuing to focus their attention. This increased memory capacity and facilitated understanding of the information presented to the students, keeping it in their memory, which is similar to the results of other studies [27,53]. Moreover, the ADIDAS model was designed according to Piaget’s cognitive constructivist theory principles and modern instructional models, which were created during COVID-19, that ensured effective learning and enhanced mental load distribution during SDL. Accordingly, the students had low cognitive load levels while learning within the ADIDAS model [43,44].

Additionally, the results show that the students taught by the ADIDAS model intend to use this learning type in the future, which agrees with other studies [28,48]. Therefore, the ADIDAS model has drawn on the interaction analysis model (IAM), which can explain why distance learning attracts students’ attention, offers an effective learning environment, and increases students’ motivation to learn the topics, all of which is compatible with other studies [27,30]. Likewise, the underlying reason that turned students’ attitudes positive may be that students came across a different education style other than the traditional SDL and interacted with their educators, peers, and content, which matches with other studies [29,47]. The ADIDAS model enabled students to engage more in an interactive learning environment with learners, content, evaluation, and technology, which is consistent with sociocultural constructivist learning theory [32,54]. Moreover, the ADIDAS model has been built according to self-regulated learning (SRL) models, which helped to direct the students’ attention selectively by providing more engaging and interactive learning elements that increased the positive attitudes among students, which is compatible with previous studies [36,37]. 

The current article includes some limitations that could be handled in future exertions, including the relationship between synchronous digital learning (SDL) sustainability and cognitive load and attitudes towards digital learning among higher education students in the KSA. This would include that the data were assembled solely from a tiny sample of students in higher education institutions in the KSA. The findings’ generalisability to elsewhere in the Gulf, the Middle East, or another geographical location should be approached with caution. Likewise, this article applied the quantitative analysis method, so future research could integrate qualitative and quantitative approaches to discover further reasons for and associations between the suggested factors. In addition, the effect of other mediating and moderating variables (students’ gender or educators’ experience, competencies, skills, digitalisation, etc.) can be combined in future research.

## 5. Conclusions

The present research developed a new instructional design model, entitled ADIDAS, and reviewed it with experts in the Arab countries’ context. The model has been provided to higher education educators as a guide for delivering SDL during emergencies, i.e., amid the COVID-19 pandemic. The model was applied for SDL amid the COVID-19 pandemic on 527 students in the colleges of KFU, KSA. The results showed that the students’ cognitive load and attitudes towards SDL were better after adopting the ADIDAS model. There were significant differences pre- and post-model adoption, and the size difference between pre- and post-model adoption was very large. The results for post-model adoption were better. The results confirmed a lower level of cognitive load and a positive attitude towards the use of SDL after the ADIDAS model. The use of instructional design models such as the ADIDAS model for SDL contributes to digital learning sustainability amid the COVID-19 pandemic.

## Figures and Tables

**Table 1:** Experts’ demographical characteristics.

Profile	Frequency	Percentage
Gender		
Male	16	47%
Female	18	53%
Country		
Egypt	18	52.943%
Jordan	7	21%
Saudi Arabia	9	26.057%
Experts’ Speciality		
Instructional technology	11	32.352%
Digital learning	4	11.764%
Cognitive psychology	9	26.473%
Information technology	10	29.411%

**Table 2:** Descriptive statistics of the model factors (*n* = 34).

Dimension	Factor	Shortcut	Items	Min.	Max.	M	S.D	VIFs	Skewness	Kurtosis
**Analyse** **(A)**	Learners (L)	A_L_1	Understand learners’ characteristics, behaviours, experiences, and skills [43,44].	4	5	4.794	0.410	4.384	−1.505	1.034
	Content (C)	A_C_1	Analyse the content into modules and determine each module’s provided time [43,44,45].	3	5	4.941	0.343	4.598	−0.514	1.248
		A_C_2	Organise the objectives and learning outcomes into knowledge, skills, and competencies [43,44,45].	4	5	4.794	0.410	4.384	−1.505	1.034
		A_C_3	Determine the interactive learning methods and activities [27,45].	4	5	4.824	0.387	4.437	−1.368	1.087
	Evaluation (E)	A_E_1	Determine the time and type of evaluation required: diagnostic, formative, structural, and collective, depending on the learner’s cognitive load and limited working memory [15,30].	4	5	4.971	0.171	4.799	−0.514	1.449
		A_E_2	Organise the assessment tools: quizzes, votes, skills observation, number of participations, self-evaluation, and peer evaluation [15,30].	4	5	4.765	0.431	4.334	−1.639	0.984
	Technology (T)	A_T_1	Determine the learning tools (platforms, hardware, and apps) that will be used synchronously and asynchronously [45,27].	4	5	4.794	0.410	4.384	−1.505	1.034
		A_T_2	Analyse the technical needs and barriers that learners may encounter [45].	4	5	4.941	0.239	4.702	−0.739	1.352
	Reviewing (R)	A_R_1	Choose the e-feedback styles during teaching [46].	3	5	4.735	0.511	4.224	−1.554	0.874
**Design** **(D)**	Learners (L)	D_L_1	Design the cognitive charts and mind maps (ABCD format) based on learners’ needs [43,44].	4	5	4.971	0.171	4.799	−0.514	1.449
	Content (C)	D_C_1	Design the lesson elements, goals, and learning outcomes [27,45].	4	5	4.794	0.410	4.384	−1.505	1.034
		D_C_2	Arrange the active learning methods and teaching strategies that will be used [27,45].	4	5	4.824	0.387	4.437	−1.368	1.087
		D_C_3	Design the interactive activities that will be presented [27,45].	4	5	4.971	0.171	4.799	−0.514	1.449
	Evaluation (E)	D_E_1	Design the assessment tools for each module (quizzes, surveys, exams, and assignments) [15,30].	4	5	4.765	0.431	4.334	−1.639	0.984
	Technology (T)	D_T_1	Design the communication groups and social networking groups, etc. [47].	4	5	4.794	0.410	4.384	−1.505	1.034
		D_T_2	Determine the technical alternatives to solve problems during the learning process [45].	4	5	4.941	0.239	4.702	−0.739	1.352
	Reviewing (R)	D_R_1	Design the feedback resources for correcting, motivating, supporting, etc. [46].	4	5	4.794	0.410	4.384	−1.505	1.034
**Improve** **(I)**	Learners (L)	I_L_1	Develop the content for communication with the learners/recipients [43,44].	3	5	4.941	0.343	4.598	−0.514	1.248
		I_L_2	Start up social networking groups [3].	4	5	4.794	0.410	4.384	−1.505	1.034
	Content (C)	I_C_1	Develop interactive activities according to the specific active learning methods and the mind maps of the learning sequence [27,45].	3	5	4.765	0.496	4.269	−1.423	0.919
		I_C_2	Organise and collect learning content into meaningful units according to educational goals and learning outcomes [27,45].	4	5	4.971	0.171	4.799	−0.514	1.449
	Evaluation (E)	I_E_1	Develop evaluation tools for each module (quizzes, surveys, exams, and assignments) [15,30].	4	5	4.765	0.431	4.334	−1.639	0.984
	Technology (T)	I_T_1	Create the communication message and ads [45].	4	5	4.794	0.410	4.384	−1.505	1.034
		I_T_2	Test the technical alternatives [45].	4	5	4.941	0.239	4.702	−0.739	1.352
	Reviewing (R)	I_R_1	Develop the feedback resources [46].	4	5	4.824	0.387	4.437	−1.368	1.087
**Do** **(D)**	Learners (L)	D_L_1	Send learners the ads and messages in the chronological order specified on the lesson map [43,44].	4	5	4.971	0.171	4.799	−0.514	1.449
		D_L_2	Record learners’ interactions during learning sessions (synchronously) and modules (asynchronously).	4	5	4.794	0.410	4.384	−1.505	1.034
	Content (C)	D_C_1	Provide synchronous educational content due to virtual classrooms and virtual platforms for asynchronous [27,45].	4	5	4.824	0.387	4.437	−1.368	1.087
		D_C_2	Utilise the interactive activities that consider the learners’ behaviour [27,45].	4	5	4.971	0.171	4.799	−0.514	1.449
		D_C_3	Make sure to use active learning methods during learning sessions and units [27,45].	4	5	4.765	0.431	4.334	−1.639	0.984
		D_C_4	Notify learners of learning resources such as websites, e-books, etc. [27,45].	4	5	4.794	0.410	4.384	−1.505	1.034
		D_C_5	Allow learners to think and work on their memories to do jobs [27].	4	5	4.941	0.239	4.702	−0.739	1.352
	Evaluation (E)	D_E_1	Provide evaluation tools (quizzes, surveys, exams, and assignments) for each session and module (do not move onto the next until completed) [15,30].	4	5	4.794	0.410	4.384	−1.505	1.034
	Technology (T)	D_T_1	Use e-learning tools and consider continuous verification of the communication [45,47].	3	5	4.941	0.343	4.598	−0.514	1.248
	Reviewing (R)	D_R_1	Provide feedback tools for each session and module (do not move onto the next until completed) [46].	4	5	4.794	0.410	4.384	−1.505	1.034
**Assess** **(A)**	Learners (L)	A_L_1	Evaluate the learners’ responses through social networks, platforms, virtual classrooms, activities, comments, answers, etc. [43,44].	4	5	4.824	0.387	4.437	−1.368	1.087
	Content (C)	A_C_1	Assess the specified mind maps, achieved goals, and learning outcomes [27,45].	4	5	4.971	0.171	4.799	−0.514	1.449
		A_C_2	Assess the learning styles and specific interactive activities [27,45].	3	5	4.706	0.524	4.182	−1.684	0.832
		A_C_3	Analyse the results from measuring the learners’ satisfaction with learning [45].	4	5	4.794	0.410	4.384	−1.505	1.034
	Evaluation (E)	A_E_1	Assess the results of the learners’ responses [15,30].	3	5	4.882	0.409	4.473	−0.862	1.123
		A_E_1	Evaluate the results from measuring the learners’ satisfaction with learning [15,30].	4	5	4.794	0.410	4.384	−1.505	1.034
		A_E_1	Assess the impact of learning by measuring the efficiency and effectiveness of each lesson [15,30].	4	5	4.971	0.171	4.799	−0.514	1.449
	Technology (T)	A_T_1	Assess the technology on the apps page to decide about continuing usage [45,47].	4	5	4.794	0.410	4.384	−1.505	1.034
	Reviewing (R)	A_R_1	Analyse the learning analytics [46].	4	5	4.824	0.387	4.437	−1.368	1.087
**Share** **(S)**	Learners (L)	S_L_1	Share the efforts of the top 10 learners during sessions or modules [43,44].	4	5	4.971	0.171	4.799	−0.514	1.449
	Content (C)	S_C_1	Record learning sessions (synchronously) and modules (asynchronously) [45].	4	5	4.765	0.431	4.334	−1.639	0.984
	Evaluation (E)	S_E_1	Share the evaluation results of assessment tools: quizzes, votes, skills observations, amount of participation, self-evaluation, and peer evaluation [15,30].	4	5	4.794	0.410	4.384	−1.505	1.034
	Technology (T)	S_T_1	Evaluate the results of e-learning tools (platforms, hardware, and apps) [45,47].	4	5	4.941	0.239	4.702	−0.739	1.352
	Reviewing (R)	S_R_1	Assess the top e-feedback styles during sessions or modules [46].	3	5	4.941	0.343	4.598	−0.514	1.248

**Table 3:** Descriptive statistics of students’ cognitive load pre- and post-ADIDAS model.

Pre ADIDAS		Cognitive Load Scale Items	Post ADIDAS	
M	SD		M	SD
		Main cognitive load		
1.403	0.573	The amount of mental effort made while learning the content of this lesson.	4.524	0.787
1.491	0.613	The amount of interaction with the elements of the content of this lesson.	4.183	0.917
1.403	0.573	The number of content items that I had to absorb at one time while learning the content of this lesson.	4.524	0.796
1.504	0.616	The amount of difficulty I experienced while learning the content of this lesson.	4.184	0.918
1.403	0.573	The extent of the interrelationship between the elements of the content of this lesson.	4.522	0.796
1.493	0.607	The average number of information contained in one paragraph in this lesson.	4.183	0.917
		Extraneous cognitive load		
1.402	0.586	The amount of stress I experienced while learning this lesson.	4.534	0.784
1.493	0.622	The number of activities not directly related to the learning task experienced while learning this lesson.	4.191	0.913
1.404	0.586	The amount of frustration I experienced while learning this lesson.	4.534	0.779
1.483	0.604	How much inconvenience did you experience while learning this lesson?	4.194	0.915
1.403	0.569	The extent of mastery in the design and organisation of the content of this lesson.	4.533	0.777
		Closely related cognitive load		
1.491	0.607	The amount of mental effort made to understand and master the content of this lesson.	4.184	0.916
1.403	0.569	The extent of involvement in learning while learning the content of this lesson.	4.532	0.784
1.494	0.607	The amount of new information was able to link to old details while learning the content of this lesson.	4.184	0.916
1.401	0.569	The motivation to understand the content of this lesson.	4.532	0.784
1.492	0.607	How well can you provide an interpretation of what you have learned?	4.184	0.921

**Table 4:** The results of paired sample *t*-test in relation to cognitive load.

Cognitive Load (CL)		M	N	SD	t	df	*P*	η2
Main CL	pre	1.453	527	0.434	−85.191	526	0.000	0.932
	post	4.354	527	0.616				
Extraneous CL	pre	1.443	527	0.436	−88.110	526	0.000	0.936
	post	4.392	527	0.604				
Closely related CL	pre	1.453	527	0.445	−80.988	526	0.000	0.925
	post	4.324	527	0.648				

**Table 5:** Descriptive statistics of students’ attitude towards SDL pre- and post-ADIDAS model.

Pre ADIDAS		Attitude towards SDL Scale Items	Post ADIDAS	
M	SD		M	SD
Knowledge development				
1.463	0.615	I find that online learning helps me learn complex concepts.	4.272	0.891
1.474	0.606	I think online learning has reduced the psychological impact of the COVID-19 pandemic.	4.283	0.885
1.472	0.618	I do not trust online learning to complete lectures during the COVID-19 pandemic	4.274	0.894
Skills development				
1.461	0.583	I see that online learning increases my interaction in lectures.	4.273	0.883
1.452	0.570	I think online learning gave me new learning skills.	4.273	0.888
1.471	0.622	I believe online learning has enabled me to learn a lot in a short time.	4.274	0.886
Larning attitudes				
1.474	0.609	I think online learning is essential and indispensable even after the COVID-19 pandemic.	4.273	0.883
1.471	0.606	Learning in a traditional classroom is better than online distance learning.	4.274	0.884
1.463	0.603	I think the online learning method is better than the traditional method, and I would like it to continue.	4.274	0.887
1.464	0.586	I enjoy the online learning experience and want it to continue.	4.282	0.893

**Table 6:** The results of paired sample *t*-test in relation to attitude towards SDL.

Attitude Towards SDL		M	N	SD	t	df	*P*	η2
Knowledge development	pre	1.473	527	0.597	−67.735	526	0.000	0.897
	post	4.272	527	0.884				
Skills development	pre	1.461	527	0.575	−67.966	526	0.000	0.897
	post	4.271	527	0.881				
Learning attitudes	pre	1.474	527	0.589	−67.294	526	0.000	0.895
	post	4.273	527	0.874				

**Figure A1:**
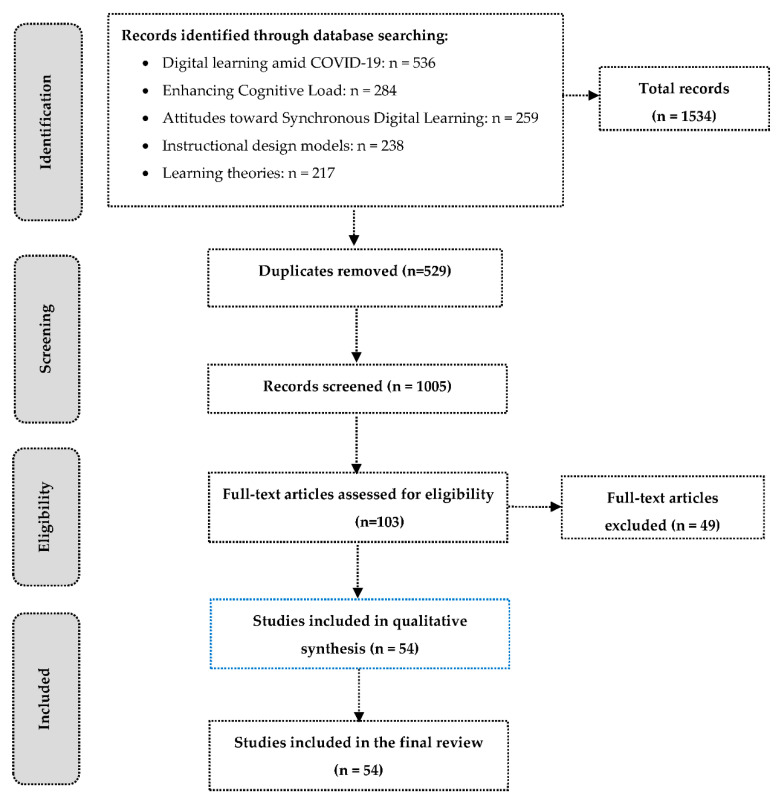
The process of literature review to develop the model.

## Data Availability

Data are available upon request from researchers who meet the eligibility criteria. Kindly contact the first author privately through email.

## Appendix A. PRISMA Flow Diagram

Inclusion/exclusion criteria: Please note that the final review included articles published in English and excluded articles published in Arabic. Additionally, it included articles related to online learning in undergraduate higher education and excluded articles about other educational grades. Likewise, it included articles that related to the ADDIE model, interaction analysis model (IAM), and models of self-regulated learning (SRL), while excluding instructional design models. Hence, it included articles on using instructional design models, while excluding others. Furthermore, it included articles on enhancing cognitive load in online learning, while excluding others. Furthermore, it included articles on cognitive load theory, Piaget’s cognitive constructivist theory, and the sociocultural constructivist learning theory, while excluding other learning theories’ articles.
